# Self and other mental health stigma among public safety professionals: a psychometric study

**DOI:** 10.1186/s41155-025-00361-2

**Published:** 2025-10-06

**Authors:** Makilim Nunes Baptista, Daniela Sacramento Zanini, Adrielli Santos de Santana, Larissa Felipe Grizza Rossi, Cristiane Faiad, Germano Gabriel Lima Esteves, Sérgio Eduardo Silva de Oliveira, Luís Gustavo do Amaral Vinha, David L. Vogel

**Affiliations:** 1 Pontifícia Universidade Católica de Campinas, Campinas, SP Brazil; 2 Pontifícia Universidade Católica de Goiás, Goiânia, GO Brazil; 3 Universidade de Brasília (UnB), Brasília, DF Brazil; 4 Department of Psychology, Iowa State University, Ames, IA USA

**Keywords:** Help-seeking, Stigma, Self-stigma, Mental health, Public safety, Professionals

## Abstract

**Background:**

Public safety professionals are frequently exposed to unique stressors and traumas in their work, which can have significant impacts on their mental well-being. However, the stigma surrounding mental health within their professional circles often deters them from seeking help when necessary. Understanding the nature and extent of this stigma is essential for developing targeted interventions to overcome barriers to help-seeking.

**Objective:**

This study aims to evaluate the psychometric properties of the Perception of Stigmatization by Others for Seeking Help Scale (PSOSH) and the Self-Stigma of Seeking Help Scale (SSOSH) among public safety professionals.

**Method:**

A total of 11,335 public safety agents from various organizations across all federal units of Brazil participated in the study, completing the PSOSH, SSOSH, and a sociodemographic questionnaire. Exploratory and confirmatory factor analyses, as well as item response theory’s graded response model, were conducted to examine the psychometric properties of the measures.

**Results:**

The validity evidence suggests one-factor structures for both instruments, with acceptable reliability coefficients. While the PSOSH demonstrated adequate psychometric properties, including good factor loadings, communalities, fit indices, reliability coefficients, and discrimination and difficulty parameters, the SSOSH yielded some results that raise questions about its psychometric properties.

**Conclusion:**

The PSOSH exhibits satisfactory psychometric quality for application in public safety contexts in Brazil. However, further investigation is needed to establish the psychometric parameters of the SSOSH more robustly.

## Introduction

 The 2024 Public Safety Yearbook reports an 8% decrease in suicide cases among civil and military police officers in Brazil, with recorded incidents declining from 137 in 2023 to 126 in 2024 (Brazilian Public Safety Forum – FBSP, 2025). Although this reduction represents progress, it must be interpreted with caution. The overall scenario indicates a decline in police fatalities, both those resulting from armed confrontations and those caused by suicide. However, a closer examination of historical data reveals a concerning trend: since 2018, suicide cases among security forces personnel have exhibited a gradual and continuous upward trajectory. This pattern stands in stark contrast to the observed decrease in intentional violent deaths (IVDs) occurring in confrontational situations. This dramatic increase may be associated with higher rates of mental health problems in recent years (Richter et al., 2019), that may have been exacerbated by the COVID-19 pandemic (Goularte et al., 2021; Santomauro et al., 2021), greater demands on public safety officers, and the limited use of psychological services, which continue to remain a taboo in modern society due to the enduring misconception that “psychologists are for those who are mentally unstable” (Luz, 2021; p. 5). Specifically, Auth et al. (2022) identified several barriers that may make it difficult for police officers to seek psychological assistance, including previous traumatic experiences related to their job, the nature of organizational interventions, and the stigma surrounding mental health care. It is noteworthy that the stigma associated with mental health care, the object of interest in this research, is one of the main factors cited in the literature for not seeking therapy in Brazil (Baptista & Zanon, 2017; Morando et al., 2018).

Two types of stigmas have been described: public other stigma and self-stigma (Corrigan, 2004). Other Public stigma (or social stigma) refers to the negative perceptions and marginalization within a social group due to various factors, such as disabilities, mental disorders, substance abuse, bodily disfigurements, stereotypical appearances, sexual orientation, or belonging to a particular race, nationality, or religious group (Corrigan, 2004; Corrigan & Watson, 2002). In turn, self-stigma arises when individuals internalize these negative perceptions, prejudice, and discrimination (Corrigan, 2004; Corrigan & Watson, 2002). Thus, individuals can begin to label their own characteristics (e.g., diagnosis of anxiety, depression) as socially unacceptable. When stigma is related to mental health, one of the most significant impacts is that the individual may try and avoid these negative perceptions (i.e., as inferior or incompetent) by not seeking treatment and thereby not having these perceptions apply to them (Vogel et al., 2025).

The effects of stigmatization on the willingness of public safety professionals, particularly police officers (civil, federal, criminal), and military professionals, to seek psychological assistance may be particularly salient (Corrigan, et al., 2012); Rodríguez, 2021). These professionals are part of a traditional organizational culture that values authority, strength, and courage. While these values can be positive and beneficial in carrying out their job duties, particularly when dealing with high-threat and risky situations (Burns 2020)), exposure to high-risk situations and events can still lead to experiencing emotional and psychological problems. In these cases, these values can also be a hindrance to seeking support as it has been suggested that values such as strength and self-sufficiency (Skopp et al., 2012) are contradictory to the act of asking for help (Karaffa & Koch, 2016; Rodríguez, 2021), which is often stigmatized as being for those that are “weak” or “out of control” (Hammer & Vogel, 2017). Similarly, there is a phenomenon identified by Reiser (1974) as the “John Wayne Syndrome” in which police officers are professionals who have control of the entire situation and who are not susceptible to illness (Rodríguez, 2021). In other words, seeking help may violate the cultural or professional “ethos,” and thus heighten the effects of both public and self-stigma as seen as a threat to job and identity (Skopp et al., 2012).

Evidence suggests that stigma, and the related concern that others would view them as not fit for duty, are some of the primary obstacles discussed as to why seeking professional care is not widely used among police officers (Jetelina et al., 2020). Karaffa and Koch (2016), for example, using samples of full-time police officers in municipal, state, and university agencies in the US states of Texas and Oklahoma, identified that public and self-stigma were negatively correlated with the demand for mental health care. Additionally, the authors found that a strong identification with police culture may lead to situations where police officers underestimate mental health issues and overlook the need to seek mental health services. Still, individuals with direct personal experience of depression or suicide strongly endorse feelings of self-stigma; those who have attempted suicide often feel ashamed and embarrassed by their behavior and tend to conceal the occurrence as much as possible (Carpiniello & Pinna, 2017). In Brazil, specifically, the difficulty for police officers to seek help from health professionals, regardless of whether it is physical or mental health, has been related to stigma-related concerns with financial loss due to removal from specific tasks, damage to career progression, and possible moral harassment (i.e., the negative view of peers regarding sick leave, prejudiced labeling, and discriminatory experiences; Cardoso & Nummer, 2018). In addition, researchers (Ricciardelli et al., 2020) have noted that among public safety professionals, there are workplace pressures that instill fears among employees about seeking time off for health treatment. As such, public safety professionals may find themselves “forced to camouflage their diagnosis” of mental health problems for fear of receiving negative performance evaluations (Ricciardelli et al., 2020). In general, these studies support the importance of further examining the role that both public and self-stigma play in the mental health help-seeking of public safety professionals in Brazil, to better be able to best support public safety professionals in need. To achieve this goal, it is crucial to further discern the extent to which stigma, whether public or self, influences these decision-making processes. For example, previous research in general populations has generally supported the idea that self-stigma is the more proximal (i.e., important) factor to focus on (see meta-analysis; Lannin and Bible, 2022 and Yu et al., 2023). However, in some populations, for example military service members, which may have some similar cultural workplace norms and values, public stigma (i.e., reactions of colleagues, concerns about impact on job/promotion) shows greater salience (Skopp et al., 2012). Furthermore, the psychometric properties of two of the most widely used measures of public stigma associated with seeking mental health service (the Perception of Stigmatization by Others for Seeking Help [PSOSH]; Vogel et al., 2009) and the self-stigma associated with seeking mental health services (the Self-Stigma of Seeking Help Scale [SSOSH]; Vogel et al., 2006), while generally shown to have sound psychometric properties across groups have shown some variation.

For example, while having been cited thousands of times in research comparing public stigma and self-stigma in various cultures (Vogel et al., 2019; Yu et al., 2023), and generally showing support for a unidimensional construct in most groups and across countries, in some groups problems with the reverse coded items (that may have more difficulty in the translation process) have been noted for the SSOSH, specifically. The authors suggested that these items might need to be removed in some populations and that researchers should consider examining the factor structure of the scale in their sample to decide whether a full or a briefer version better fits their data (Vogel et al., 2025). They also noted that cultural factors and individual differences may influence the way that people interpret the construct and complete scale items. As such, comparing stigma across cultures may require adaptation of the measurement.

Given this context, the present study aims to evaluate construct validity of the PSOSH and SSOSH among Brazilian public safety professionals. The psychometric investigation of these measures is a primary and fundamental step to guarantee their use in the specific context of Brazilian public security. It is necessary to examine the construct’s structure, the scores’ reliability, and the parameters of discrimination and difficulty of the items to make valid and safe applications and interpretations of these measures. Our specific aims are to examine the internal structure of these two measures in a Brazilian sample of public safety professionals, assess the measures’ reliability, and ultimately scrutinize item discrimination and difficulty parameters utilizing the graduated response model (Samejima, 1969).

## Method

### Participants

This research enlisted the participation of 11,335 public safety agents from various agencies, encompassing the Military Police, Civil Police, Criminal Police, Technical-Scientific Police, and Military Fire Brigade, spanning across all Federative Units in Brazil. Furthermore, there was the participation of Federal institutions such as the Federal Highway Police and the National Penitentiary Department (currently the National Secretariat for Penal Policies). This study utilized a non-probabilistic convenience sample. The participants had an average age of 40.77 years (SD = 7.90) and had worked at their respective institutions for an average of 14.11 years (SD = 8.40). Table 1 depicts additional sociodemographic information. As can be seen, most of the sample contained men (81.7%), married (73.3%), from Brazil’s southeast region (52.2%) and with (monthly) family income between R$3300.01 and R$7700.00 (89.1%).

**Table 1:** Sociodemographic data of the sample

Sociodemographic information	*N*	%
Region of residence		
North	61	9.40
Northeast	2411	21.10
Midwest	853	7.50
Southeast	5906	52.20
South	1104	9.80
Sex		
Male	9261	81.70
Female	2024	17.90
Did not inform	50	0.40
Marital status		
Married/stable union	8307	73.30
Single	2118	18.70
Widower	42	0.40
Divorced	868	7.70
(Monthly) Family income		
Up to R$ 1100.00 (low income)	43	0.60
From R$ 1100.01 to R$ 2200.00 (lower-middle income)	129	1.80
From R$ 2200.01 to R$ 3300.00 (middle income)	619	8.50
From R$ 3300.01 to R$ 5500.00 (upper-middle income)	3648	50.30
From R$ 5500.01 to R$ 7700.00 (high income)	2810	38.80

### Instruments

#### Sociodemographic questionnaire

We developed a questionnaire to gather data from research participants to discern characteristics including gender, age, geographical region, marital status, and family income.

#### Public stigma

The *Perception of Stigmatization by Others for Seeking Help* (PSOSH; Vogel et al., 2009) scale is a concise questionnaire comprising five items designed to assess an individual's perception of the potential prejudices or stigmatization from those in their immediate social circle if they were to seek psychological assistance (referred to as other public stigma). For instance, one item asks participants to rate the degree to which they believe others would view them less favorably for seeking help (e.g., “Thinking of you in a less favorable way”). Items are rated on a five-point Likert scale ranging from “not at all” (1) to “very much” (5). Raw scores on the PSOSH range from 5 to 25 points, with higher scores indicating a greater perception of stigmatization by third parties for seeking psychological help. The initial investigation into the internal structure of the PSOSH was conducted by Baptista et al. (2016) among Brazilian university students. This study revealed the scale's unidimensionality, with a single factor explaining 62.9% of the item’s variance. The internal consistency coefficient observed by the Authors was 0.84, using the Cronbach’s alpha method.

#### Self-stigma

The *Self-Stigma of Seeking Help* (SSOSH; Vogel et al., 2007) scale is a 10-item instrument designed to evaluate an individual's response when recognizing the necessity of seeking psychological assistance. For instance, one item asks participants to rate their reaction to the idea of seeking help, such as feeling out of adjustment (e.g., “I would feel out of adjustment if I sought psychological help”). Items are rated on a five-point Likert scale, ranging from 1 “completely disagree” to 5 “completely agree.” Five items are reverse scored so that total scores range from 10 to 50 points, with high scores indicating a high level of internalized stigmatizing perceptions about seeking psychological help. The instrument was adapted for Brazil by Baptista and Zanon (2017) who observed, in a sample of Brazilian university students, an internal consistency coefficient of 0.73, using the Cronbach’s alpha method.

### Procedures

The present study is part of the research entitled “Assessment of Health and Intervention Propositions inthe Public Security Area – a National Study” carried out throughout Brazilian territory through a Decentralized Execution Term (TED No. 009/2019/CGPP/DPSP/SENASP) between the University of Brasília (UnB) and the Ministry of Justice/National Public Security Department (MJ/SENASP). The research was approved by an Ethics Committee (Approval number: nº 3,965,395). Due to the secrecy, confidentiality, and sensitivity of the information, data collection was carried out through an online platform developed specifically for the research, identified as the Public Safety Health Assessment System (SASSP). Furthermore, the National Public Safety Secretariat (SENASP) provided, on a confidential basis, a list containing participants’ institutional email addresses and WhatsApp contact numbers to the project coordinators. The participants were informed that the responses were anonymous, and the data were handled without identification. Thus, participants received the invitation to the study in three ways: a link sent by researchers to the institutional email address, a link sent by the institutions themselves to the participants’ email address and/or WhatsApp. When accessing the research, participants had access to the Free and Informed Consent Form (FICF). After accepting the FICF, participants responded to the research protocol.

### Data analysis

Descriptive statistics, including frequency, percentage, mean, and standard deviation, were employed to analyze the sample profile using IBM SPSS version 24. Subsequently, the sample was randomly divided into two subsamples for further analysis. Exploratory factor analysis (EFA) and confirmatory factor analysis (CFA) were conducted to identify evidence of validity based on the internal structure of the instruments. In the first subsample (*n* = 5667), the R language (version 4.0.2) was utilized along with the psych package (Revelle & Revelle, 2015) to investigate the optimal number of factors to be extracted via parallel analysis (PA; Horn, 1965). Subsequently, after determining the appropriate number of factors, exploratory factor analyses (EFAs) were performed for both instruments. The EFAs utilized the polychoric correlation matrix and employed the weighted least squares (WLS) estimation method, which is recommended for ordinal variables. Additionally, Promax rotation was employed to accommodate potential correlations between factors, as it accounts for the independence of factors (Damásio & Dutra, 2017; Kiers, 1997). To obtain adequate structural validity indicators of the scales, factor loadings above 0.30 in the EFA and communalities above 0.50 were expected (Matos & Rodrigues, 2019). Subsequently, to confirm the adequacy of the model identified in the EFA for both instruments, CFAs were conducted with the second subsample (*n* = 5668) using the weighted least square mean and variance adjusted (WLSMV) estimation method (Li, 2016). The model fit was assessed using the root mean square error of approximation (RMSEA), Comparative Fit Index (CFI), and Tucker-Lewis Index (TLI) indices. These indices indicate a good fit when the RMSEA is below 0.08 and the CFI and TLI are above 0.95 (Brown, 2015; Kenny et al. (2015); Mair, 2018). Thus, with evidence from the factorial structure of the PSOSH and SSOSH and using the total sample (*n* = 11,335), it was also decided to evaluate the parameters of discrimination and difficulty of the items using the graduated response model (Samejima, 1969), using the R language (v. 4.0.2) through the mirt package (Chalmers et al., 2015). Finally, also with the total sample, the psych package (Revelle & Revelle, 2015) in the R language (v. 4.0.2) was used to evaluate the reliability of the PSOSH and SSOSH scores through the alpha coefficients of Cronbach (α), Guttman's lambda 6 (λGuttman6) and McDonald's omega (ω), assuming adequate reliability for internal consistency coefficients equal to or greater than 0.70.

## Results

**Fig. 1:**
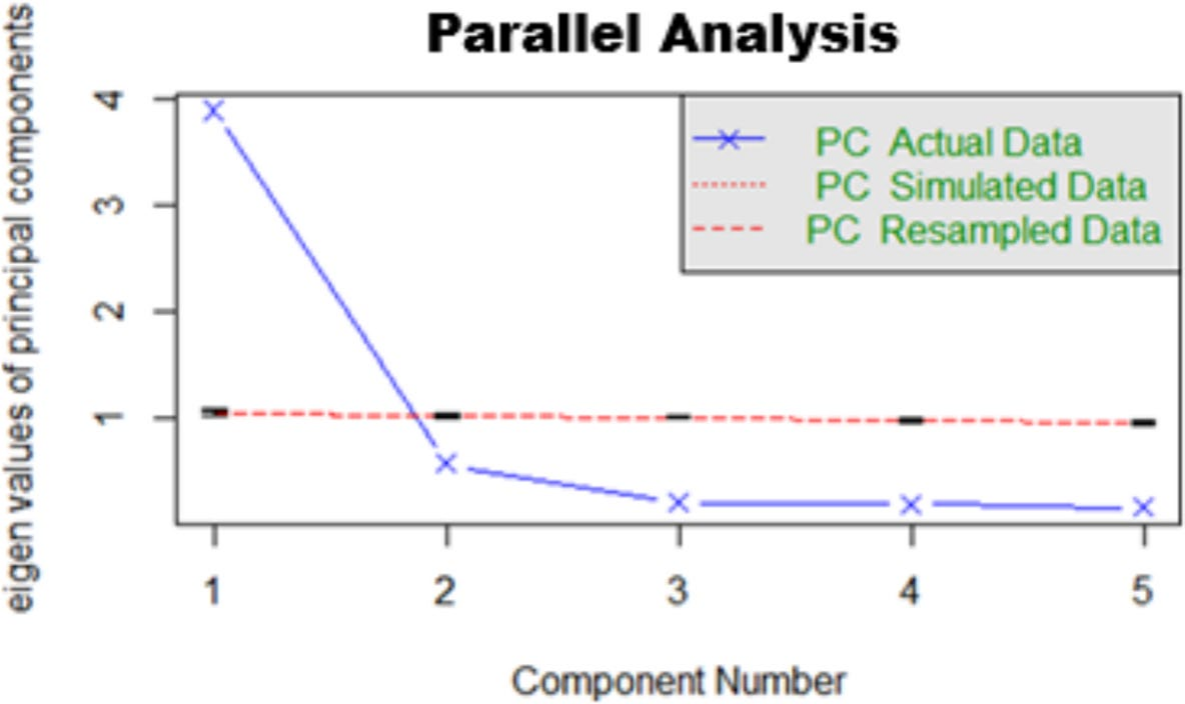
PSOSH parallel analysis

Figure 1 indicates that the PA of the PSOSH demonstrated a single-factor structure. As it is a short scale with only five items, the unidimensional premise was confirmed through the PA. Furthermore, the analysis of the factor loadings obtained through EFA confirmed that all items are highly related to the construct (see results in Table 2).

**Table 2:** PSOSH exploratory and confirmatory factor analyses

Item	EFA λ	Communality	CFA λ
1. Reacting negatively to you	0.73	0.53	0.70
2. Thinking bad things about you	0.87	0.76	0.86
3. Thinking that you have serious disorders	0.90	0.82	0.87
4. Thinking of you in a less favorable way	0.90	0.81	0.94
5. Thinking that you could be a risk to others	0.85	0.72	0.83

*EFA *exploratory factor analysis, *CFA *confirmatory factor analysis,* λ* factor loading

Table 2 exhibits all factor loadings greater than 0.70 and communalities greater than 0.50. With this factorial structure, a CFA was carried out, which demonstrated fit indices of CFI = 0.97, TLI = 0.95, and RMSEA = 0.24, in addition to an explained variance of 73%. It is important to note that the elevated RMSEA value (0.24) results from the strong inter-item correlations (> 0.70) observed among Items 2, 4, and 5, which reduce the model’s effective degrees of freedom and inflate the approximation-error estimate, even when the model is correctly specified, an effect previously reported for unidimensional models with redundant indicators or low residual variance (Chen, et al., 2008); McNeish & Wolf, 2023; Kenny et al., 2015; Taasoobshirazi & Wang, 2016). This pattern may also signal a lack of true unidimensionality, warranting the exploration of alternative specifications such as allowing correlated latent factors or modeling correlated error terms (Muthén 2004)).

Based on these results, and guided by the modification indices, two error covariances were added: one between items 1 and 2, and another between items 3 and 5. Additionally, the diagonally weighted least squares (DWLS) estimator was used. The results indicated a better fitting model: CFI = 1.000, TLI = 1.000, and RMSEA = 0.030. Moreover, the scale’s reliability approached 0.90 across all three methods, underscoring its consistency and dependability. (*α* = 0.90; ω = 0.93; λ^6^ de Guttman = 0.89).

Table 3 shows the difficulty of the items (thresholds) between the scale’s response points, as well as the items’ discrimination parameters. According to the data obtained through item response theory (IRT), it can be affirmed that item 5 demands the highest level of the trait to be endorsed, followed by item 3, and that all items were highly discriminative.

**Table 3:** PSOSH IRT parameters

Item	*b1*	*b2*	*b3*	*b4*	*a*
1. Reacting negatively to you	− 0.43	0.35	1.08	2.23	2.11
2. Thinking bad things about you	− 0.36	0.19	0.77	2.05	3.71
3. Thinking that you have serious disorders	0.06	0.54	1.18	2.29	2.91
4. Thinking of you in a less favorable way	− 0.36	0.21	0.79	1.87	3.70
5. Thinking that you could be a risk to others	0.40	0.86	1.58	2.65	2.21

*a *discrimination parameter, *b1-4 * difficulty threshold parameter

**Fig. 2:**
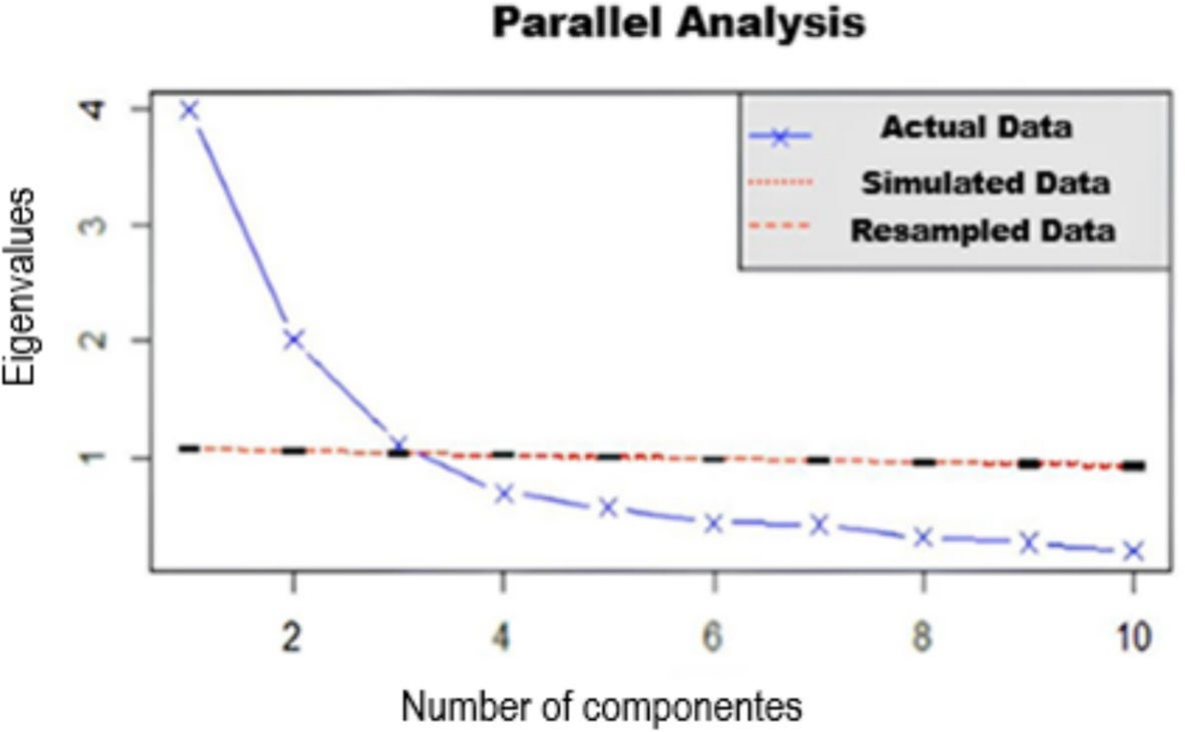
SSOSH parallel analysis

Concerning SSOSH, the PA initially demonstrated the existence of three factors, as displayed in Fig. 2. However, the third factor was very close to the simulated eigenvalue in the PA, so the two-factor model was investigated.

The two-factor model was tested; however, the model only separated items with positive content (e.g., “My self-esteem would increase if I spoke to a therapist.”) and those with negative content (e.g., “I would feel out of place if I sought psychological help”). This result can be due to a possible method bias in statistical analysis. Therefore, both EFA and CFA were carried out with just one factor, as initially proposed for the original version of this measure. Thus, an EFA was initially carried out. Item 5 was loaded below 0.30 and was then eliminated. The results regarding the remaining nine items are expressed in Table 4.

**Table 4:** SSOSH exploratory and confirmatory factor analyses

Item	Factor loadings–EFA	Communality	Factor loadings–CFA
6	0.89	0.79	0.92
8	0.79	0.62	0.82
3	0.79	0.61	0.84
1	0.70	0.48	0.73
7	− 0.56	0.31	− 0.41
2	− 0.44	0.19	− 0.30
9	− 0.44	0.19	− 0.30
10	0.35	0.12	0.37
4	− 0.30	0.09	− 0.16

*EFA* exploratory factor analysis, *CFA* confirmatory factor analysis

This factor accounted for 38% of the variance among the items, with two items loading up to 0.40 and six items exhibiting communalities below 0.50. Upon conducting CFA and introducing covariance terms between the errors of items 2, 4, 7, and 9, the following indices were obtained: CFI = 0.96, TLI = 0.94, and RMSEA = 0.09. The reliability of the scale presented acceptable indices (*α* = 0.73; ω = 0.82; λ^6^ de Guttman = 0.76). Although the RMSEA value of 0.09 is somewhat above the commonly recommended threshold (< 0.08), it can still be interpreted as marginally acceptable (Fabrigar et al., 1999). Moreover, simulation studies have shown that models with few degrees of freedom often yield inflated RMSEA values, even when correctly specified (Kenny et al., 2015). In contrast, the obtained values of CFI = 0.96 and TLI = 0.94 are indicative of excellent fit, confirming that the model fits the data well when evaluated holistically. In this context, we concluded that an RMSEA of 0.09 is acceptable, provided it is interpreted in conjunction with global fit indices, in accordance with the recommendations of Kenny et al. (2015).

Regarding IRT, as shown in Table 5, all 10 items were evaluated, showing that the most difficult items were 3 and 6, respectively. In terms of discrimination, some items were well below expectations, such as items 4 and 5; four items exhibited low discrimination (10, 2, 7, and 9), one item showed moderate discrimination (1), and three items demonstrated high and/or very high discrimination (2, 6, and 8) (Baker, 2001).

**Table 5:** SSOSH IRT parameters

Item	*b1*	*b2*	*b3*	*b4*	*a*
1	0.36	1.00	1.62	2.85	1.68
2	− 0.18	0.86	1.58	2.43	0.82
3	1.05	1.47	2.17	2.64	2.14
4	− 3.53	− 0.59	2.14	3.55	0.50
5	− 2.49	− 0.47	2.17	4.58	0.37
6	0.78	1.18	1.74	2.33	3.60
7	− 0.50	0.63	2.09	2.84	1.07
8	0.48	0.96	1.87	2.65	2.34
9	− 0.49	0.51	1.73	2.77	0.85
10	− 1.30	− 0.15	0.82	3.13	0.67

*a *discrimination parameter, *b1-4 *difficulty threshold parameter

## Discussion

Despite the availability of evidence-based treatments, many public safety professionals in Brazil in need of mental health care do not seek help (Auth et al. (2022). One of the most cited reasons for not seeking help is the stigma associated with seeking such care (Baptista & Zanon, 2017; Morando et al., 2018). The PSOSH (Vogel et al., 2009) and the SSOSH (Vogel et al., 2007) were designed to measure public and self-stigma, respectively, but the scales were developed in the USA, with a general sample of young adults, and so their applicability among other groups such as public safety professionals in Brazil is not known. Therefore, this research aimed to evaluate the psychometric properties of the PSOSH and SSOSH in a sample of public safety professionals in Brazil to examine the evidence of validity based on the internal structure, analyze the degree of reliability of these measures and, finally, analyze the parameters of discrimination and difficulty of the items through the graduated response model. Such an examination is needed for researchers and professionals to be able to evaluate the factors driving individuals’ stigma-related reluctance to seek mental health care prior to treatment as well as during treatment to help mitigate dropout (Britt et al., 2015; Hoge et al., 2014; Jennings et al., 2016) and maximize treatment effects (Kulesza et al., 2017; Ociskova et al., 2018).

The results suggest that the PSOSH (Vogel et al., 2009) presents evidence of validity and reliability for estimating the other public stigma that public safety professionals have in relation to seeking mental health services.

Specifically, it was observed that PSOSH presented a single-factor structure consistent with extant literature (Vogel et al., 2009). Overall, this result underscores how public stigma can hinder individuals from seeking specialized help, as they fear it may alter how they are perceived by those within their social circle (Nascimento & Leão, 2019). Building on this, this was the first study to use IRT to examine the PSOSH, and in the IRT analysis, it became apparent that on the PSOSH scale, items 5 (“Thinking that you could be a risk to others”) and 3 (“Thinking that you have serious disorders”) exhibited the highest rates of the evaluated trait. These items present serious value judgments that would have a greater impact on social life, compared to the others (e.g., 1—reacting negatively to you; 2—thinking bad things about you; 4—thinking of you in a less favorable way). In this regard, these items appear to indicate the obstacles to seeking help identified by Cardoso and Nummer (2018), notably the worry regarding peers’ perception that those who need help and take time off to do are seriously disturbed.

The validity results for the SSOSH (Vogel et al., 2007) were a little less straightforward. While previous research with young adult populations in the USA and many other areas of the world (e.g., Vogel et al., 2019), including Brazil (Baptista et al., 2016), has generally suggested a single factor structure, the initial results in the current study suggested that SSOSH may present a two-factor structure. It seems, however, this division may have been the result of semantic considerations (i.e., potential method bias) with the items segregating based on positive or negative wording. This is consistent with the developers of the SSOSH scale noting that in some samples, the negative wording may be more difficult to respond to, particularly after translation (Vogel et al., 2025). They found that if this method factor was modeled (i.e., controlled for in the analyses) or if the 5 negatively worded items were dropped, then the expected unidimensional structure was maintained (Vogel et al., 2019). This was also found to be generally true for the current sample, and so for future research in public safety professionals in Brazil, it is recommended to consider either directly examining the factor structure of the scale, before analyses, or to simply use a briefer version in their work (see Vogel et al., 2025), as briefer versions (3- and 7-items) have been found to fully assess the construct, provide similar predictive ability, and remove the potential method effects (see Brenner et al., 2021).

Also worthy of noting is that in the current other IRT analyses, the three positively worded items that best reflected the latent trait (“I would feel inadequate if I went to a therapist for psychological help”; “Seeking psychological help would make me feel less intelligent”; “I would feel inferior if I sought help from a therapist”), were consistent with the items identified in the one previous IRT analysis of the SSOSH (Brenner et al., 2021). These three items most strongly reflect the idea of comparing themselves with others. In the context of public safety professionals, these items seem to represent the elements that might be most likely to contrast with the organization's cultural values and the values associated with the John Wayne Syndrome (Reiser, 1974). For instance, there is often a perception that individuals in roles like police officers cannot afford to appear weak or acknowledge emotional challenges (Burns 2020); Newell et al., 2022; Rodriguez, 2021).

## Implications

Several important implications arise from the findings of this study. First, this work supports the growing body of research noting the importance of public and self-stigma in the decision to seek help around the world (Vogel et al., 2013, 2007; Yu et al., 2023) and for specific populations (i.e., police: Grupe, 2023; military: Skopp et al., 2012). While some variation in the measurement of self-stigma was noted and needs to be further examined in future work, both public and self-stigma were generally found to have adequate psychometric properties. As such, this work has implications for future researchers who may want to continue to examine the role of these stigmas in creating barriers to seeking help for public safety professionals. This work could focus on (1) the longitudinal effects of public and self-stigma on help-seeking behaviors, (2) examining the direct role of organizational values on stigma, (3) using the scales as a screeners at intake to help determine if concerns about public and/or self-stigma could lead to early drop out or limited treatment compliance, or (4) assessing the effectiveness of an intervention in reducing stigma among public safety professionals.

Specifically, the current findings give some focus on the types of interventions that may be best to reduce the stigma of seeking help among public safety professionals. Previous researchers (e.g., Hammer & Vogel, 2010) found that a brochure focused on normalizing and reframing messaging about therapy in line with a group’s values can reduce self-reported stigmatizing beliefs. For public safety professionals in Brazil, this study found that public stigmatizing messages such as “being a risk to others” and “having a serious disorder” were most salient, while for self-stigma, the beliefs that I would be “inadequate, inferior, and less intelligent” were most reflective of the underlying construct. Thus, interventions that reframe these beliefs by normalizing mental health and portraying help-seeking as an act of strength to help oneself and those around them (Schreiber & Hartrick, 2002) and to present mental health problems as resolvable (Mann & Himelein, 2004) may have the greatest impact. These interventions could be reflected in a few ways (e.g., brochures, online messaging, outreach/workshops) to talk about stigma in public safety organizations. However, direct outreach/workshops might be particularly needed to change organizational values. Work with military groups, for example, has noted the perceptions of the messages coming from their chain of command and from peers were more salient than in other populations, and thus the organizational leadership and/or peers may need to be involved if changes in stigmatization are to take hold (Skopp et al., 2012).

## Limitations and conclusion

Having measures of public and self-stigma with robust psychometric properties for the public safety context is extremely valuable, as it offers a resource to identify individuals who are resistant to seeking help from mental health professionals. Public safety professionals experience highly stressful jobs, and having all available resources, including therapy, available to assist them is important. Such measures could be used to assess interventions to reduce stigma and increase willingness to seek mental health assistance, thereby alleviating the unnecessary suffering and potentially mitigating the risk of suicide among public safety professionals (Baptista & Zanon, 2017). The psychometric adequacy of PSOSH and SSOSH can boost research on the assessment of other stigma in the context of public safety and its relationship with other aspects related to the health of these professionals, such as post-traumatic stress disorder (PTSD) and depression (Sousa et al., 2022).

While it is worth highlighting that this study evaluated a significant sample of public safety professionals (more than 11,000) distributed across the five regions of Brazil, there still were some limitations to the study. The descriptive analysis reveals that most public safety professionals in the sample were young adults, married, with children, and predominantly male. Consequently, it is plausible that the influence of this profile may have influenced the responses obtained, potentially impacting the item parameters. Furthermore, another possible limitation is the impact of the data collection method, which involved computer-mediated, remote, and asynchronous procedures. This approach might have favored the selection of participants with a higher affinity for computers and electronic devices. Moreover, there is the possibility that when answering the survey, the participants were not alone, which may have influenced the answers in some way. Nevertheless, it is notable that findings from a meta-analytic study indicate that self-report surveys administered via traditional paper-and-pencil methods or computer-based platforms generally yield comparable results (Weigold et al., 2018).

The results presented in the current study indicate a significant step forward in the assessment of psychological aspects within the public safety context. Our findings provide scientific evidence regarding the validity and reliability of instruments designed for evaluating stigma to be used with professionals of public safety institutions. As such, this work lays the foundation for future studies to continue to explore various aspects of the measures, including examining its invariance across gender or types of safety forces (e.g., police officers and firefighters). Furthermore, new studies may delve into broader aspects of the assessment of stigma in this population, such as its potential risk for suicidal behavior, absenteeism, and mental health problems.

## Data Availability

The datasets generated during and/or analyzed during the current study are not available due to confidentiality issues related to public security.

## Abbreviations

PSOSH: Perception of Stigmatization by Others for Seeking Help Scale
SSOSH: Self-Stigma of Seeking Help Scale
USA: United States of America
SASSP: Public Safety Health Assessment System
SENASP: National Public Safety Secretariat
FICF: Free and informed consent form
EFA: Exploratory factor analysis
CFA: Confirmatory factor analysis
PA: Parallel analysis
WLS: Weighted least squares
WLSMV: Weighted least square mean and variance adjusted
RMSEA: Root mean square error of approximation
CFI: Comparative Fit Index
TLI: Tucker Lewis Index
IRT: Item response theory
